# Genome characterization of cluster EE microbacteriophage Trackstar

**DOI:** 10.1128/mra.00406-26

**Published:** 2026-07-22

**Authors:** Jake Medina, Fidel Calderon Iglesias, Emmanuel Mirabal, Mauricio Montero, Patricia Montesino Rivas, Jeferson Roca, Elizabet Rodriguez, Jordy Zambrano, Lara Becerra-Reymundo, Jaime Mayoral, Patricia Waikel

**Affiliations:** 1 Department of Biological Sciences, Florida International University 5450, Miami, Florida, USA; Queens College Department of Biology, Queens, New York, USA

**Keywords:** bacteriophage genetics

## Abstract

Trackstar was isolated using *Microbacterium folioru*m NRRL B-24224 in Miami, Florida. Its genome is 17,033 bp with 24 predicted protein-coding genes and 69.0% guanine–cytosine (GC) content. Trackstar has a siphoviral morphology and is assigned to cluster EE based on gene content similarity.

## ANNOUNCEMENT

Bacteriophages are abundant and diverse (1), shape microbial communities, influence bacterial evolution, and have applications in industrial, agricultural, and medical contexts (2). We isolated and characterized the novel microbacteriophage Trackstar to increase the understanding of bacteriophage diversity and function.

**Fig 1:**
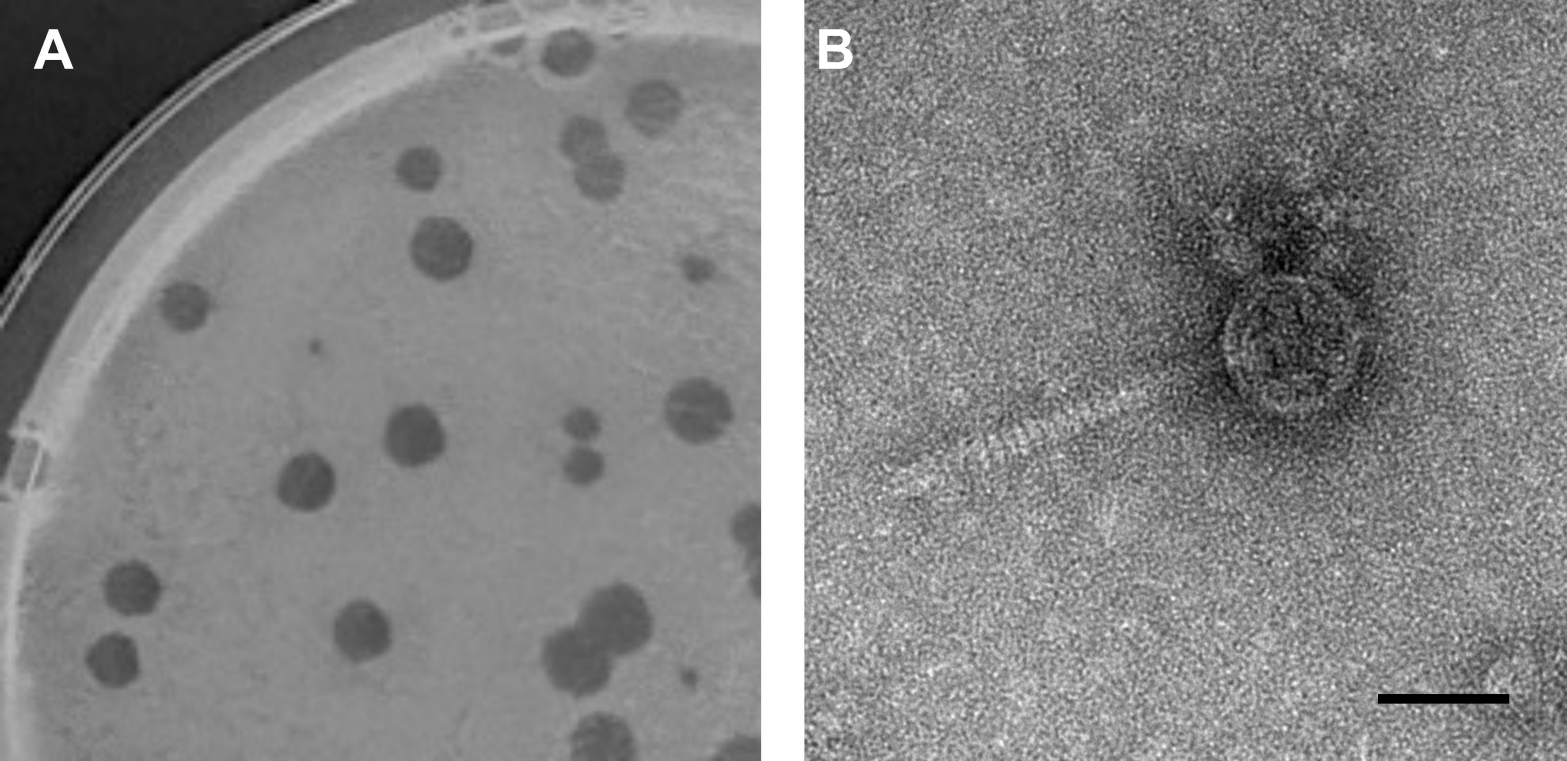
(**A**) Plaque assay of bacteriophage Trackstar showing clear 4.21 ± 0.44 plaques (*N* = 10) formed on *Microbacterium foliorum* NRRL B-24224. Plate is 80 mm in diameter. (**B**) Transmission electron micrograph of bacteriophage Trackstar demonstrating siphoviral morphology, including an icosahedral capsid 47.12 ± 0.60 nm in diameter and short noncontractile tail 118.33 ± 1.20 nm in length (*N* = 3). Scale bar: 50 nm. Phage samples were placed on 200-mesh formvar-covered, carbon-coated copper grids (EMS, Hatfield, PA, USA), incubated for 1 min, rinsed with ultrapure water, and stained with 2% uranyl acetate. Images were taken at 100 kV using a Hitachi HT7800 120kV TEM with an AMT NanoSprint15B digital camera.

Trackstar was isolated from an enriched soil sample collected in Miami, Florida (25.70536 N, 80.37412 W) using *Microbacterium foliorum* strain NRRL B-24224 as a host. Soil was suspended in peptone-yeast-calcium (PYCa) medium, incubated for 2 h, then centrifuged at 2,000 *g*. Supernatant was filtered through a 0.22-µm filter, inoculated with *M. foliorum*, and incubated at 30°C for 2 days to enrich for phages infecting *M. foliorum*. The enriched sample was filtered and plated in PYCa top agar with *M. foliorum*. Individual plaques were purified through two rounds of plating. Trackstar formed large, clear plaques 4.21 ± 0.44 mm (*N* = 10) in diameter (Fig. 1A). Transmission electron microscopy revealed a siphoviral morphology, with a capsid measuring 47.12 ± 0.60 nm and a flexible, noncontractile tail of 118.33 ± 1.20 nm (*N* = 3) (Fig. 1B).

Trackstar’s dsDNA was extracted from phage particles in lysate using the *Quick*-DNA Viral Kit (Zymo). Libraries were constructed using the NEBNext Ultra II DNA Library Prep Kit and sequenced on an Illumina NextSeq 1000 (XLEAP-P1 kit), generating 4,198,097 single-end 100-bp reads (about 23,593× coverage). Raw reads were trimmed with cutadapt 4.7 (using the option: –nextseq-trim 30) and filtered with skewer 0.2.2 (using the options: -q 20 -Q 30 -n -l 50) prior to assembly (3, 4). Raw reads were then assembled using Consed v29 (5) and Unicycler v0.5.0 (6), producing a 17,033-bp genome with 69.0% guanine–cytosine (GC) content. Genome ends were determined as previously reported (7). Trackstar was assigned to cluster EE based on 35% or higher gene content similarity to other phages in PhagesDB (8).

The genome was annotated using DNA Master v5.23.6, Glimmer v3.2 (9), GeneMark v3.25 (10), Starterator v1.2 (https://github.com/SEA-PHAGES/starterator), Phamerator v3.0 (11), BLASTp (12) using the National Center for Biotechnology Information (NCBI) non-redundant and PhagesDB databases, HHpred (13) (using PDB_mmCIF70, Pfam-A, UniProt-SwissProt, NCBI Conserved Domains databases), and PECAAN v.20241104 (https://blog.kbrinsgd.org/). Default settings were used unless otherwise noted.

Trackstar’s genome contains 17,033 bp with 24 predicted genes and exhibits the compact organization characteristic of EE cluster phages, with genes encoding structural, assembly, and lysis functions representing the primary genomic content (14). A −1 translational frameshift was identified at 7,378 bp near the 3′ end of gp10 at a conserved slippery sequence (GGGAAAC), producing a tail assembly chaperone fusion protein. Programmed translational frameshifting has been reported in small, tailed bacteriophages and contributes to virion assembly and structural diversity (14, 15). Trackstar’s minor tail protein (gp13) is smaller than in many EE cluster phages and present in only a subset of EE genomes (~16%), suggesting divergence in tail architecture that may influence host interactions. Two genes were predicted to encode membrane proteins based on DeepTMHMM (16). No tRNA or tmRNAs were identified using ARAGORN v1.2.41 (17) or tRNAscan-SE v2.0 (18). Trackstar lacks recognizable integrase or immunity repressor genes, indicating it is unlikely to establish lysogeny.

## Data Availability

Trackstar is available at GenBank accession no. PV876924 and Sequence Read Archive (SRA) no. SRR33237341.
